# Eccentric-Oriented Strength Training in Anterior Cruciate Ligament Rehabilitation: A Scoping Review

**DOI:** 10.3390/medicina62061109

**Published:** 2026-06-07

**Authors:** Boris Žigmund, Erika Zemková

**Affiliations:** Department of Biological and Medical Sciences, Faculty of Physical Education and Sports, Comenius University in Bratislava, 81469 Bratislava, Slovakia; erika.zemkova@uniba.sk

**Keywords:** ACL rehabilitation, eccentric training, eccentric exercise

## Abstract

*Background and Objectives:* Persistent quadriceps weakness, muscle atrophy, and functional deficits are common following anterior cruciate ligament (ACL) reconstruction and may compromise return to sport and increase the risk of reinjury. Eccentric-oriented strength training has been widely used to enhance muscle strength and hypertrophy in various musculoskeletal conditions; however, its specific application within ACL rehabilitation remains insufficiently explored. The aim of this scoping review was to map the existing evidence on the use of eccentric-oriented strength training in ACL rehabilitation, identify gaps in the current literature, and provide suggestions for future research. *Materials and Methods:* A scoping review search was conducted in PubMed, Scopus, Web of Science, and PEDro from inception to February 2026 using the following keywords and Boolean operators: (“anterior cruciate ligament”, “ACL”, “anterior cruciate ligament reconstruction”, “ACLR”) AND (“eccentric training”, “eccentric exercise”, “eccentric loading”, “flywheel training”, “isoinertial training”). Eligible studies included studies that investigated eccentric exercises as part of ACL rehabilitation and reported outcomes related to muscle strength, muscle morphology, functional performance, or return to sport. Data were extracted and synthesized descriptively in accordance with the PRISMA-ScR extension for Scoping Reviews guidelines. Methodological quality and risk of bias were evaluated using the PEDro scale (RCTs) and the ROBINS-I tool (non-randomized studies). *Results:* Fifteen studies met the inclusion criteria. The included literature primarily examined isokinetic eccentric exercise, eccentric cycling, early progressive eccentric resistance training, Nordic hamstring exercise, eccentric ergometry, and flywheel strength training. Most studies reported improvements in quadriceps strength and muscle morphology, with additional benefits observed in functional performance measures (i.e., hop tests), gait mechanics, and limb symmetry. Evidence was unevenly distributed across rehabilitation phases, with relatively few studies focusing on the mid-phase of ACL rehabilitation. *Conclusions:* Eccentric-oriented strength training represents a promising but underexplored component of ACL rehabilitation. However, the existing literature lacks standardized protocols, comprehensive outcome measures, and phase-specific guidance, particularly during the mid and late stages of rehabilitation. Further high-quality studies are needed to clarify the optimal timing, dosage, and integration of eccentric training across rehabilitation phases.

## 1. Introduction

Anterior cruciate ligament (ACL) injury is one of the most common injuries in sports involving rapid pivoting, changes of direction, and deceleration [1]. Deficits in force production and force–velocity characteristics during eccentric muscle actions can persist long after injury and may remain present even at return to sport [2]. Although ACL reconstruction (ACLR) is widely regarded as a safe and effective procedure [3,4], important limitations and unresolved challenges in rehabilitation outcomes remain. Despite structured rehabilitation programs, many individuals continue to exhibit persistent deficits in quadriceps and hamstring strength, reduced rate of force development (RFD), muscle atrophy, interlimb asymmetry, and impaired neuromuscular control. These findings are common after ACL reconstruction and have been associated with impaired functional performance and increased risk of reinjury [5,6]. These deficits can persist for months to years after surgery and are associated with impaired functional performance, delayed return to sports, and an increased risk of re-injury or development of post-traumatic osteoarthritis [7,8].

Several clinical and experimental studies suggest that traditional rehabilitation protocols, which predominantly emphasize concentric or submaximal strength training, may be insufficient to fully restore muscle function and interlimb symmetry [9,10]. Mounting evidence indicates that neural deficits are likely an important contributing factor to increased injury risk [11].

Eccentric training is an effective tool in ACL rehabilitation because it allows for greater muscle force production than concentric training. During eccentric contractions, muscles resist high external loads, providing a substantial mechanical stimulus to muscle fibers, tendons, and connective tissues [2]. A review by Zhong et al. [12] suggested that eccentric training with accentuated loading is associated with specific neural and mechanical adaptations, including increased recruitment of high-threshold motor units, enhanced tendon stiffness, and an improved ability to absorb and generate force. Adaptations may contribute to improvements in the rate of force development and the efficiency of the stretch-shortening cycle. Eccentric resistance training leads to a range of neuromuscular adaptations that go beyond simple increases in muscle size or maximal strength. Previous research [13] has shown that eccentric loading can enhance activity in the motor cortex, improve motor unit recruitment and firing behavior, and reduce inhibitory mechanisms within the neuromuscular system. These changes indicate that eccentric training may alter how the nervous system regulates force production. In anterior cruciate ligament rehabilitation, where deficits in neuromuscular control and explosive strength are common, eccentric training may play a key role in restoring function and supporting a safe return to sport. Further evidence supporting the role of eccentric training in neuromuscular performance comes from the study by Stasinaki et al. [14], who demonstrated that fast-velocity eccentric resistance training leads to significant improvements in rate of force development (RFD), with these changes being partly explained by increases in muscle fascicle length. In contrast, slower eccentric loading appears to preferentially induce hypertrophic adaptations without meaningful improvements in rapid force production.

These findings highlight that eccentric training adaptations are highly velocity-specific, with fast eccentric contractions playing a key role in enhancing explosive neuromuscular performance, while slower contractions are more closely associated with structural muscle adaptations. In that context, a recent meta-analysis [15] by Zhongzhong et al. demonstrated that flywheel resistance training may produce greater improvements in muscle power compared to traditional resistance training in healthy individuals, while no significant differences were observed in maximal strength development in healthy populations.

Current evidence has examined the role of eccentric training in musculoskeletal rehabilitation; however, no comprehensive synthesis currently describes its application across the different phases of ACL rehabilitation. There is limited guidance regarding the timing, progression, and modality-specific implementation of eccentric training in practice.

This scoping review was conducted to map the existing evidence on the use of eccentric-oriented strength training in anterior cruciate ligament rehabilitation, identify gaps in the current literature, and provide suggestions for future research.

## 2. Materials and Methods

### 2.1. Study Design

Due to substantial heterogeneity in study designs and intervention characteristics, a scoping review was adopted to provide an overview of how eccentric training is implemented in ACL rehabilitation and to identify gaps in the current evidence. This scoping review was conducted in accordance with the PRISMA extension for Scoping Reviews (PRISMA ScR) guidelines [16].

### 2.2. Search Strategy

A literature search was performed in PubMed, Scopus, and Web of Science from inception to February 2026. In addition, the PEDro (Physiotherapy Evidence Database) was searched to identify relevant randomized controlled trials in physiotherapy. The final search was conducted on 22 February 2026. The search strategy combined terms using Boolean operators. The Boolean operator AND was used to combine different conceptual domains, while OR was used to include synonymous terms. The full electronic search strategy for PubMed was as follows: (“anterior cruciate ligament”[Title/Abstract] OR “ACL”[Title/Abstract] OR “anterior cruciate ligament reconstruction”[Title/Abstract] OR “ACLR”[Title/Abstract]) AND (“eccentric training”[Title/Abstract] OR “eccentric exercise”[Title/Abstract] OR “eccentric strength training”[Title/Abstract] OR “eccentric loading”[Title/Abstract] OR “flywheel training”[Title/Abstract] OR “isoinertial training”[Title/Abstract]). Only articles published in English were considered. Reference lists of included articles were also screened to identify additional relevant studies. The full search strategies for all databases are provided in the Supplementary Material.

### 2.3. Eligibility Criteria

Studies were included if they involved participants with ACL reconstruction or ACL injury and investigated eccentric exercises as part of rehabilitation. Eligible studies reported outcomes related to muscle strength, muscle morphology, functional performance, or return to sport. Only original research articles and clinical studies published in English were considered, including randomized controlled trials, non-randomized studies, clinical trials, and case reports. Non-randomized studies were defined as studies in which participants were allocated to interventions without random assignment, including prospective cohort and quasi-experimental designs. Studies were excluded if they did not include an eccentric exercise component; were animal or in vitro studies; were conference abstracts, editorials, or opinion papers; or did not focus on ACL rehabilitation. The inclusion criteria were defined to ensure that only studies directly relevant to ACL rehabilitation and eccentric training were included, allowing for a focused mapping of the available evidence.

### 2.4. Protocol and Registration

No protocol was registered for this scoping review, which represents a methodological limitation. This is consistent with the exploratory nature of scoping reviews, which aim to map the existing evidence rather than evaluate intervention effectiveness. However, this scoping review was retrospectively registered on the Open Science Framework (https://doi.org/10.17605/OSF.IO/MR83J).

### 2.5. Study Selection

**Figure 1:**
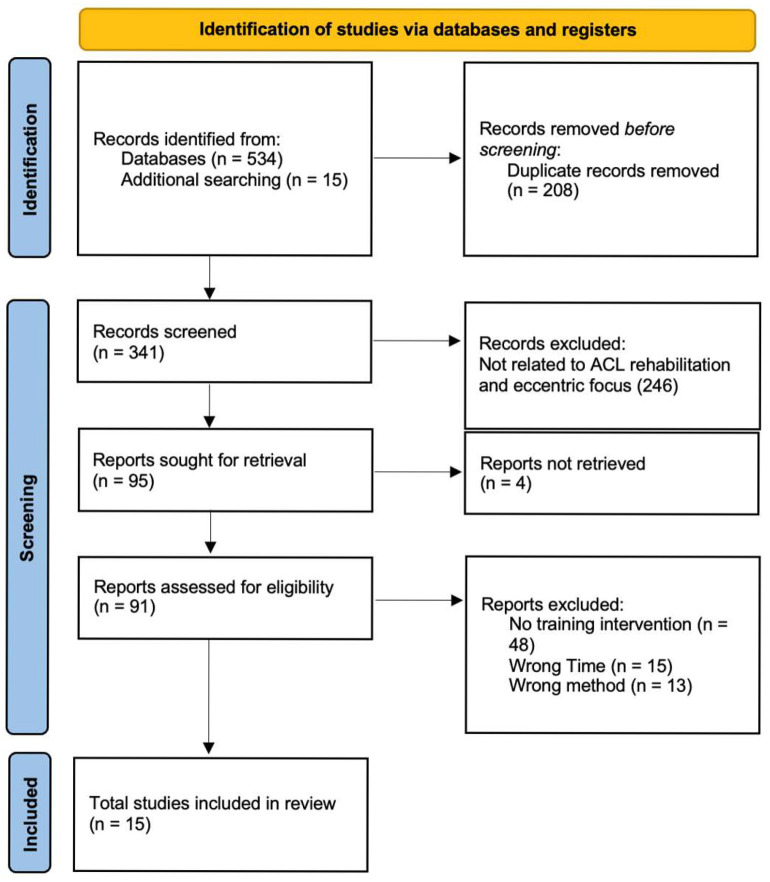
PRISMA ScR flow diagram.

All records identified through database searching were exported, and duplicates were removed. Titles and abstracts were screened for relevance, followed by full-text assessment based on the eligibility criteria. The study selection process is illustrated in the PRISMA ScR flow diagram Figure 1. Two reviewers independently screened titles, abstracts, and full-text articles for eligibility in accordance with predefined inclusion criteria. Any disagreements were resolved through discussion.

### 2.6. Data Charting

A standardized data extraction form was developed and refined through an initial pilot phase on a small subset of studies to ensure consistency and relevance of collected data. Relevant data were extracted from the included studies, including study characteristics, participant details, intervention type, duration, outcome measures, and key findings. Rehabilitation phase classification was based on the time since surgery as reported in each included study. Studies were categorized as follows: early stage (0–12 weeks post-surgery), mid-stage (3–6 months post-surgery), and late stage (>6 months post-surgery or return-to-sport phase). The rehabilitation stages (early, mid, and late) were categorized according to commonly used postoperative rehabilitation timelines and functional recovery goals reported in the ACL rehabilitation literature [3,4,6]. When the exact time from surgery was not explicitly stated, classification was based on the rehabilitation stage or goals described by the authors. Data extraction was conducted by one reviewer and independently verified by a second reviewer. Any disagreements were resolved through discussion.

### 2.7. Data Synthesis

Due to heterogeneity in study designs and outcome measures, a quantitative meta-analysis was not performed. The extracted data were synthesized descriptively and organized into predefined thematic categories based on the phase of rehabilitation (early, mid, and late) and the type of eccentric intervention. Studies were compared narratively within and across these categories, focusing on intervention characteristics, outcome measures, and key findings. This approach enabled a structured comparison of findings across studies and the identification of research gaps.

### 2.8. Quality Assessment and Critical Appraisal

A critical appraisal was conducted to assess the methodological quality of the included studies and to support the interpretation of the findings. The methodological quality of randomized controlled trials (RCTs) was assessed using the Physiotherapy Evidence Database (PEDro) scale. The PEDro scale consists of 11 items, of which 10 contribute to the final score, evaluating internal validity and statistical reporting. Each item is scored as either present (1) or absent (0), resulting in a total score ranging from 0 to 10. Higher scores indicate better methodological quality. Studies scoring 6 or higher were of moderate-to-high quality. The assessment was performed independently by the authors, and any discrepancies were resolved through discussion. The risk of bias in non-randomized studies was assessed using the ROBINS-I (Risk of Bias in Non-Randomized studies of Interventions) tool. This tool evaluates bias across seven domains: confounding, selection of participants, classification of interventions, deviations from intended interventions, missing data, measurement of outcomes, and selection of reported results. Each domain was rated as low, moderate, serious, or critical in terms of risk of bias. An overall risk-of-bias rating was assigned based on the highest level of bias identified across domains. The assessment was conducted independently, and disagreements were resolved by consensus. The methodological quality and risk-of-bias assessments were performed by one reviewer and independently verified by a second reviewer, with disagreements resolved through discussion.

## 3. Results

### 3.1. Search Results

The database search identified 534 records, and an additional 15 records were identified. After removing duplicates, 341 records remained for screening. Following title and abstract screening, 246 records were excluded because they were not related to anterior cruciate ligament rehabilitation or an eccentric rehabilitation focus. Thus, 95 full-text articles were assessed for eligibility. Of these, 76 were excluded because they lacked an eccentric training component. In total, 15 studies were included in this scoping review. A summary of included studies on eccentric-oriented strength training in ACL rehabilitation is outlined in Table 1 and Table 2 [17,18,19,20,21,22,23,24,25,26,27,28,29,30,31].

The included studies were published between 2006 and February 2026. Out of the 15 included studies, nine studies were randomized controlled trials (RCTs) [18,20,21,22,23,25,28,29,31], five were non-randomized studies (Non-RCTs) [17,19,26,27,30], and one was a case report [24]. Across the included literature, eccentric training was delivered using different approaches, including isokinetic eccentric exercise [17,18,26,30]; eccentric cycling [21]; feasibility of Nordic hamstring exercise after hamstring graft [28]; early progressive eccentric resistance training [25]; eccentric ergometry [24]; combination of eccentric, concentric, and plyometric training [22,23]; flywheel strength training [19,20,29,31]; and combinations of eccentric exercise with neuromuscular electrical stimulation [27]. Intervention duration ranged from four to twelve weeks, with some studies reporting longer follow-up periods [28]. Functional performance outcomes, including hop tests and return-to-sport test criteria, were assessed in six studies [18,19,21,22,23,29]. Additional outcomes included muscle strength (quadriceps and hamstrings) [17,18,19,20,22,23,24,25,26,27,29,30,31], gait biomechanics [21,26], and balance or limb symmetry [22,23]. Measures of the rate of force development (RFD) were reported in one study [19], while patient-reported outcomes (e.g., KOOS, IKDC, TSK-CF) were also assessed in two studies [22,23]. The sample size varied markedly, ranging from a single participant to 96 participants in the randomized controlled trial. Included populations were predominantly young adults, with mean ages generally between 20 and 30 years [17,18,22,23,29], although some studies reported wider age ranges [25]. Sex distribution differed across studies, with several investigations including only male participants [18,21,23,24,26,30], one study focusing exclusively on female athletes [22], and some including mixed samples [17,19,20,25,27,28,29,31]. There was considerable variability in the timing of eccentric training following ACL reconstruction. In some studies, the intervention was initiated early in the rehabilitation process, as soon as 2–6 weeks postoperatively [18,20,24,25,27,31], whereas in others, it was introduced later, ranging from approximately 8 weeks to 3 months post-surgery [17,21,22,23], 4–6 months postoperatively [29], or 6 months and more [19,26,28,30]. Both hamstring tendon autografts [17,18,20,21,28] and bone–patellar tendon–bone (BPTB) grafts [22,23,26,29,30] were represented. Two studies included mixed graft populations [19,25], while graft type was not reported in one case [31].

Table 3 presents the distribution of included studies across different rehabilitation phases following ACL rehabilitation. A total of six studies were categorized in the early phase (0–12 weeks), focusing primarily on quadriceps activation, prevention of muscle atrophy, and early controlled eccentric loading using modalities such as isokinetic training [18], ergometry [24,25], neuromuscular electrical stimulation [27], and inertial eccentric training [20,31]. Four studies were categorized in the mid-phase (3–6 months), where interventions increasingly targeted quadriceps and hamstring strength using an isokinetic eccentric program [17], combined with eccentric–plyometric training approaches [22,23]. Functional performance outcomes, including hop tests, were also commonly assessed in this phase. Five studies were categorized in the late phase (>6 months/return-to-sport phase). These studies primarily examined return-to-sport criteria, functional symmetry, and performance outcomes, with interventions including flywheel training [19,29] or isokinetic training [26,30], Nordic hamstring exercises, and eccentric cycle ergometer training [21].

Table 4 summarizes the characteristics of eccentric training protocols. Training volume varied markedly between studies, from low-volume protocols such as single-set training to failure to more structured multi-set programs (e.g., 3–4 sets of 8–12 repetitions). Similarly, loading strategies differed substantially, including isokinetic protocols with fixed angular velocities, flywheel-based eccentric overload using varying inertial loads, percentage-based loading relative to the uninvolved limb, and bodyweight-based exercises such as the Nordic hamstring exercise.

### 3.2. Quality and Risk-of-Bias Assessment

The methodological quality of the included randomized controlled trials, as assessed using the PEDro scale, is presented in Table 5. All studies reported random allocation of participants, while allocation concealment was present only in a subset of trials. Baseline comparability between groups was achieved across all studies. None of the included studies implemented blinding of participants or therapists, which is expected given the nature of exercise-based interventions. In contrast, blinding of outcome assessors was reported in the majority of studies. All studies demonstrated adequate follow-up rates exceeding 85% and consistently reported between-group comparisons and measures of variability. However, intention-to-treat analysis was not reported in any of the included trials. Overall, the included RCTs demonstrated moderate methodological quality, with PEDro scores ranging from 6 to 7 out of 10. These findings should be interpreted cautiously due to limited blinding procedures, absence of intention-to-treat analyses, small sample sizes, and the predominance of non-randomized designs.

The risk-of-bias assessment of non-randomized studies is presented in Table 6. Overall, the included studies demonstrated a moderate-to-high risk of bias. The primary sources of bias were related to confounding and lack of randomization. Most studies [17,19,26,27] showed moderate risk in participant selection and deviations from intended interventions. Measurement of outcomes and reporting bias were generally rated as low to moderate. One study [30] was classified as having a high overall risk of bias, while the remaining studies demonstrated a moderate overall risk.

## 4. Discussion

The findings of this scoping review indicate that eccentric training is applied across different phases of ACL rehabilitation using a wide range of modalities, with isokinetic eccentric training being the most frequently used approach. Other modalities, including flywheel training, early progressive eccentric loading, and combined eccentric–plyometric interventions, were less commonly reported [17,18,22,29,30,31]. The available evidence indicates that eccentric interventions have been implemented at different stages of rehabilitation, with some studies [18,24,25,27,31] introducing eccentric loading in the early or middle postoperative phase and others focusing on later-stage strengthening [26,28,29,30]. The mapped evidence indicates that eccentric training interventions in ACL rehabilitation are primarily concentrated in early postoperative strengthening and late-stage rehabilitation, whereas the mid-phase of rehabilitation remains insufficiently investigated. Most studies primarily focused on quadriceps and hamstring strength and hop performance. Lepley et al. [27] reported that the combination of NMES with eccentric exercises approximately six weeks post-surgery resulted in improvements in quadriceps strength and neuromuscular activation compared with NMES alone. Patra et al. [31] implemented an early flywheel-based eccentric training program following ACL reconstruction, which was associated with improvements in eccentric power and muscular endurance compared with standard rehabilitation. Stojanović et al. [29] applied a progressively structured flywheel training program over six weeks, increasing training volume while maintaining a consistently high load across all participants, and reported improvements in countermovement jump height, single-leg vertical jump, hop tests, and isometric strength compared with traditional resistance training. Henderson et al. [19] demonstrated that, although athletes presented symmetrical maximal strength prior to the intervention, deficits in rate of force development were still evident. Naczk et al. [20] demonstrated that progressive flywheel-based eccentric training initiated during the early postoperative phase was associated with improvements in muscle strength and interlimb symmetry, although no additional benefits were observed in balance or isokinetic strength. Flywheel-based eccentric training has been investigated across different stages of ACL rehabilitation; however, the available evidence remains limited and heterogeneous. Existing studies have applied flywheel interventions at varying time points following surgery, including early postoperative phases and late-stage return-to-sport phases. Across these studies, substantial variability is evident in the applied protocols, including differences in inertial loads, exercise selection, training volume, frequency, and progression strategies. This lack of standardization makes direct comparison between studies difficult and limits the ability to determine optimal training parameters. In addition, Kasmi et al. [22,23] demonstrated that eccentric and combined eccentric–plyometric training reported improvements in functional performance and stability measures in elite male and female athletes following ACL reconstruction. These findings suggest that eccentric loading may be particularly relevant during the mid-stage of rehabilitation, when neuromuscular capacity and sport-specific readiness are progressively restored. Most interventions used isotonic or isokinetic exercise modalities [17,18,22,23,24,25,26,27,30]. During the mid-to-late rehabilitation phase, several studies reported improvements in quadriceps and hamstring strength and functional performance following isokinetic eccentric interventions [17,18,25,26,30]. Some studies also reported improvements in return-to-sport rates following eccentric isokinetic programs. However, these findings are not directly comparable due to differences in the study design, training protocols, and outcome measures. While Vidmar et al. [18] compared two eccentric loading modalities and primarily reported strength outcomes, Ong et al. [17] compared eccentric and concentric rehabilitation approaches and included functional hop tests. Despite similar loading parameters, the reported outcomes differed substantially. Brasileiro et al. [30] demonstrated improvements in muscle strength and neuromuscular activation, while Coury et al. [26] reported alterations in knee biomechanics, including increased valgus during gait. This suggests that although eccentric isokinetic training effectively enhances muscular capacity, its effects on movement quality may be more complex and not uniformly beneficial. It is important to interpret these findings with caution, as the studies used non-randomized designs with small sample sizes, which increases the risk of bias and limits the ability to establish causal relationships. The limited use of blinding procedures, the absence of intention-to-treat analyses, and the predominance of relatively small cohorts further reduce the overall certainty and clinical generalizability of the available evidence. In contrast, mechanical variables such as rate of force development (RFD) or eccentric RFD, braking impulse, strength of other muscle groups (gluteal, adductor, and calf), and force–time metrics are rarely assessed [19], even though ACL injury mechanisms are predominantly eccentric in nature and involve high demands on rapid force absorption and deceleration capacity. This review highlights that eccentric training interventions following ACL reconstruction exhibit substantial heterogeneity. Eccentric training is not applied as a standardized intervention but rather as a broad category encompassing multiple distinct approaches with different physiological targets. As a result, direct comparison between studies remains limited, and it is difficult to identify optimal training parameters for clinical practice.

All included interventions were categorized as eccentric-oriented training approaches; however, important conceptual differences exist between the applied modalities. Traditional eccentric resistance training and isokinetic eccentric training primarily focus on controlled lengthening contractions and isolated strength development, whereas flywheel and eccentric overload interventions aim to increase eccentric loading demands through inertial resistance mechanisms and may provide greater neuromuscular and force–power stimuli. In contrast, eccentric–plyometric approaches additionally target the stretch-shortening cycle, reactive strength, and sport-specific neuromuscular performance, whereas eccentric ergometry and eccentric–concentric cycling are more commonly used during earlier rehabilitation stages to promote muscle activation and progressive loading with lower joint stress. Therefore, these modalities may involve partially distinct physiological mechanisms, neuromuscular adaptations, and rehabilitation objectives, making direct comparisons between studies difficult.

A key limitation of the existing literature is the lack of clearly defined, standardized protocols for volume, intensity, progression, and exercise selection. This variability is evident across different rehabilitation phases, where eccentric loading is inconsistently implemented, particularly in the mid-stage. The limited integration of eccentric loading during this phase may contribute to persistent deficits in force absorption and impaired readiness for return to sport. Variables more closely related to injury mechanisms, such as rate of force development (RFD), eccentric impulse, and force–time characteristics, are rarely assessed. This represents a fundamental gap, as ACL injury mechanisms are strongly associated with rapid force absorption and deceleration capacity, which are not adequately captured by conventional strength measures. Additionally, the contribution of other muscle groups involved in force absorption, including the gluteal, adductor, and calf muscles, is rarely examined. Some evidence suggests that eccentric training may induce modality-specific adaptations. For example, early inclusion of inertial eccentric-overload training did not consistently demonstrate additional gains in isokinetic strength or balance, but it contributed to improved morphological recovery, particularly through increased muscle mass of the operated limb and improved interlimb symmetry. These findings suggest that eccentric training may influence structural and neuromuscular recovery differently depending on the applied modality and rehabilitation phase.

From a methodological perspective, the moderate quality of randomized controlled trials and the moderate-to-high risk of bias observed in non-randomized studies weaken the overall strength of the available evidence. Therefore, the current evidence should be interpreted as exploratory rather than confirmatory. Most research has been conducted in male populations, while female and youth athletes—despite their higher ACL injury risk—remain underrepresented. Long-term outcomes, including reinjury rates, joint health, and post-traumatic osteoarthritis, are also rarely evaluated. Despite the growing body of evidence, important limitations remain. There is insufficient high-quality randomized evidence directly comparing flywheel-based interventions with conventional eccentric or isokinetic approaches. Consequently, clear recommendations for isolated eccentric-oriented training remain lacking, as the literature provides inconsistent guidance on dosage, progression, and integration within a comprehensive rehabilitation framework.

From a practical perspective, the included eccentric modalities appear to target partially different rehabilitation objectives. Flywheel and eccentric-overload interventions were more commonly associated with improvements in rapid force production, interlimb symmetry, and hop tests, although substantial heterogeneity existed between studies regarding inertial loads, exercise selection, training frequency, progression strategies, and rehabilitation timing. Traditional isokinetic eccentric approaches primarily focus on isolated strength restoration and controlled loading progression at fixed angular velocities but may place lower sport-specific neuromuscular demands on the athlete. Combined eccentric–plyometric interventions may further enhance reactive strength and stretch-shortening cycle function; however, these approaches entail greater mechanical demands and may be less appropriate during earlier rehabilitation stages. Eccentric ergometry and eccentric–concentric cycling were more commonly used during earlier rehabilitation phases to facilitate progressive loading and muscle activation while minimizing joint stress.

This review also has several limitations. First, the inclusion of heterogeneous study designs and interventions limits direct comparison between studies. Second, the methodological quality of the included studies was moderate, with several studies presenting a moderate-to-high risk of bias. Additionally, the limited use of blinding procedures, the absence of intention-to-treat analyses, relatively small cohorts, and the inclusion of several non-randomized studies with moderate-to-high risk of bias further reduce the overall certainty and clinical generalizability of the available evidence. The lack of standardized reporting of outcomes, particularly eccentric-specific variables, limits the interpretation and comparability of findings across studies.

Future research should focus on well-designed, phase-specific studies with clearly defined loading parameters and standardized outcome measures in order to establish evidence-based guidelines for the integration of eccentric training into ACL rehabilitation protocols. Such standardization is essential to improve comparability between studies and to enhance the practical applicability of eccentric training within clinical practice.

## 5. Conclusions

This scoping review revealed that eccentric training is increasingly incorporated into ACL rehabilitation. However, its application remains inconsistent and insufficiently standardized across rehabilitation phases. The available evidence suggests potential benefits for strength, functional performance, and morphological recovery, but it is limited by heterogeneity in study design, training protocols, and outcome measures. Importantly, key neuromuscular variables relevant to ACL injury mechanisms remain underexplored. Therefore, current findings should be interpreted cautiously, as this review maps existing evidence rather than confirms clinical superiority. Future research should prioritize high-quality, phase-specific trials with clearly defined loading strategies and comprehensive outcome assessment to support evidence-based implementation of eccentric training in clinical practice.

## Figures and Tables

**Table 1:** Summary of included studies on eccentric-oriented strength training in ACL rehabilitation.

Author (Year)	Study Design	Eccentric Modality	Duration	Main Outcomes	Key Findings
Ong et al. (2024) [17]	Non-RCT—prospective cohort study	Isokinetic eccentric exercise	6 weeks	Isokinetic peak torque, functional tests	Improved strength and functional recovery
Vidmar et al. (2020) [18]	RCT	Isokinetic eccentric vs. constant-load eccentric	6 weeks	Quadriceps isometric and eccentric isokinetic strength, hypertrophy, hop tests	Isokinetic eccentric training reported improvements
Milandri & Sivarasu (2021) [21]	RCT	Eccentric vs. concentric cycling	6–8 weeks	Strength, gait biomechanics	Eccentric cycling was associated with greater improvements
Kasmi et al. (2021) [22]	RCT	Eccentric vs. plyometric vs. combined training	6 weeks	Stability, knee function, RSI, hop test	Combined eccentric–plyometric training was associated with improvements in stability and functional performance
Gerber et al. (2007) [25]	RCT	Early progressive eccentric exercise	12 weeks	Muscle size, strength, function	Increased hypertrophy and strength
Brasileiro et al. (2011) [30]	Non-RCT—clinical trial	Eccentric quadriceps training	12 weeks	Muscle morphology, strength	Increased muscle volume and strength
Coury et al. (2006) [26]	Non-RCT	Isokinetic eccentric training	6 weeks	Gait kinematics	Improved knee kinematics
Lepley et al. (2014) [27]	Non-RCT	Eccentric + NMES	6 weeks	Quadriceps function	Combined therapy was associated with improved quadriceps function
Norte et al. (2025) [28]	RCT	Nordic hamstring exercise	4 weeks	Hamstring strength	Feasible and improved hamstring strength
Gerber et al. (2006) [24]	Case report	Eccentric ergometry (negative work)	Early phase	Strength	Safe and feasible
Stojanović et al. (2023) [29]	RCT	Flywheel vs. traditional	6–8 weeks	Strength, hop tests, RTS criteria	Reported improvements in strength and a higher proportion meeting RTS criteria
Patra et al. (2025) [31]	RCT	Flywheel vs. traditional	6 weeks	Strength, strength endurance, isometric strength, balance	Was associated with improvements in eccentric strength and endurance strength
Henderson et al. (2022) [19]	Non-RCT	Flywheel Bulgarian squat	8 weeks	RFD, MVIC, CAR	Was associated with RFD (early force production); MVIC improved mainly in weaker individuals; no change in CAR
Kasmi et al. (2023) [23]	RCT	Eccentric + plyometric training (COMB)	6 weeks	Isokinetic peak torque, KOOS, IKDC, TSK-CF	Combined training was associated with improvements in strength and psychological outcomes compared to eccentric or plyometric alone
Naczk et al. (2026) [20]	RCT	Inertial (eccentric overload, flywheel) training	12 weeks	Isokinetic strength, inertial strength, muscle mass, balance	Improved interlimb symmetry and muscle mass; no additional effect on isokinetic strength vs. standard rehab

Abbreviations: RCT, randomized controlled trial; Non-RCT, non-randomized controlled trial; IKDC, International Knee Documentation Committee; KOOS, Knee Injury and Osteoarthritis Outcome Score; TSK-CF, Tampa Scale for Kinesiophobia—Cardiac Form; RFD, rate of force development; MVIC, maximal voluntary isometric contraction; CAR, central activation ratio; NMES, neuromuscular electrical stimulation; RTS, return to sport.

**Table 2:** Summary of studies’ outcomes.

Author (Year)	Study Design	*n* (Groups)	Age (Mean ± SD)	Sex	Time After ACLR	Graft Type
Ong et al. (2024) [17]	Non-RCT prospective cohort study	EXP: 18/CON: 18	EXP: 26.3 ± 6.7; CON: 25.6 ± 4.3	EXP: 14 M 4 F, CON: 17 M, 1 F	18.8 ± 3.1 vs. 19.9 ± 3.4 weeks	Hamstring
Vidmar et al. (2020) [18]	RCT	EXP: 15/CON 15	EXP: 26.9 ± 5.8; CON: 24.3 ± 4.6	Male only	~6 weeks (start), ~3 months (post-test)	Hamstring
Milandri & Sivarasu (2021) [21]	RCT	30 EXP: 15/CON: 15	EXP: 37.4 ± 10.7 CON: 28.6 ± 6.8	Male only	10–16 weeks	Hamstring
Kasmi et al. (2021) [22]	RCT	40 (10/10/10/10)	20.3 ± 3.2	Female only	14 weeks	BPTB
Gerber et al. (2007) [25]	RCT	EXP: 20/CON: 20	18–50 years (range)	Mixed	3 weeks (start)	HT + BTB
Brasileiro et al. (2011) [30]	Non-RCT—clinical trial	9	31.3 ± 5.8	Male only	9.4 ± 0.7 months	BPTB
Coury et al. (2006) [26]	Non-RCT	EXP: 5/CON: 10	32 ± 7.8	Male only	9 ± 1.3 months	BPTB
Lepley et al. (2014) [27]	Non-RCT	36 EXP/10 CON	21.9 ± 4.9	17 F/29 M	6 days (NMES), 6 weeks (ECC start)	BPTB + HT
Norte et al. (2025) [28]	RCT	EXP: 19/CON: 6	EXP: 22.8 ± 3.0; CON: 21.8 ± 3.3	14 F/11 M	49.4 ± 26.5 vs. 43.2 ± 23.1 months	Hamstring
Gerber et al. (2006) [24]	Case report	1	26	Male only	3 weeks	HT -> BPTB revision
Stojanović et al. (2023) [29]	RCT	22 (EXP: 11/CON: 11)	19.9 ± 4.4	14 M/8 F	5–6 months	BPTB
Patra et al. (2025) [31]	RCT	96 (EXP: 47/CON: 49)	NR	88 M/8 F	3 weeks	NR
Henderson et al. (2022) [19]	Non-RCT	11	20.8 ± 2.7	Mixed	546 ± 308 days	HT + BPTB + QT
Kasmi et al. (2023) [23]	RCT	40 (10/10/10/10)	20.3 ± 3	Male only	~14 weeks	BPTB
Naczk et al. (2026) [20]	RCT	24 (12/12)	36.7 ± 11.1	5 F/19 M	2 weeks (baseline), from week 7	Hamstring (ST + gracilis)

Abbreviations: ACLR, anterior cruciate ligament reconstruction; BPTB, bone–patellar tendon–bone; HT, hamstring tendon; QT, quadriceps tendon; EXP, experimental group; CON, control group; NMES, neuromuscular electrical stimulation; NR, not reported; M, male; F, female.

**Table 3:** Distribution of included studies across rehabilitation phases following ACL reconstruction.

Rehabilitation Phase	Included Studies	Focus in Studies	What Is Still Missing
Early (0–12 weeks)	Gerber et al. 2006 [24]; Gerber et al. 2007 [25]; Vidmar et al. (2020) [18]; Lepley et al. 2014 [27]; Patra et al. 2025 [31]; Naczk et al. 2026 [20]	Quadriceps activation; prevention of muscle atrophy; graft-related muscle deficits; controlled eccentric loading (isokinetic, ergometry, NMES)	Limited assessment of functional force production; minimal evaluation of multi-joint strength; absence of kinetic or performance-based measures
Mid (3–6 months)	Ong et al. 2024 [17]; Kasmi et al. 2021 [22]; Kasmi et al. 2023 [23]; Milandri & Sivarasu 2021 [21]	Quadriceps and hamstring strength; introduction of eccentric cycling and combined eccentric–plyometric training; hop performance	Limited evaluation of other muscle groups (calf, hip abductors/adductors, gluteus muscles); lack of comprehensive strength profiling; limited jump mechanics and force-time analysis
Late (>6 months/RTS/post-rehabilitation)	Coury et al. 2006 [26]; Stojanović et al. 2023 [29]; Norte et al. 2025 [28]; Brasileiro et al. 2011 [30]; Henderson et al. 2022 [19]	Return-to-sport criteria; flywheel training; Nordic hamstring; functional symmetry and performance	Very limited number of flywheel studies; scarce prospective data on reinjury risk; limited biomechanical and longitudinal outcomes

**Table 4:** Detailed characteristics of eccentric training protocols (volume, intensity, and progression) in ACL rehabilitation.

Study	Phase	Training Type	Frequency	Volume (Sets × Reps)	Load/Intensity	Progression
Gerber et al. (2007) [25]	Early (3 weeks)	Progressive eccentric (negative work)	3–5×/week	3–4 sets × 8–12 reps	High-load eccentric (supramaximal possible)	Gradual increase in resistance over 12 weeks
Gerber et al. (2006) [24]	Early (3 weeks)	Eccentric ergometry	3–5×/week	5–30 min/session	Progressive negative work (ergometer)	Duration + intensity increased
Lepley et al. (2014) [27]	Early (~6 weeks)	Eccentric + NMES	3×/week	3–4 sets × 8–12 reps	~60–70% MVC + NMES	Constant load, neural stimulus via NMES
Patra et al. (2025) [31]	Early (3 weeks)	Flywheel (squat, lunge)	2×/week	3 × 10 per exercise	0.02 → progressive inertia	Weekly increase (0.005–0.01 kg·m^2^)
Vidmar et al. (2020) [18]	Early–mid	Isokinetic eccentric	2×/week	3–4 sets × 10 reps	Controlled velocity	Constant velocity
Naczk et al. (2026) [20]	Early–mid (~7 weeks)	Flywheel	2–3×/week	Not fixed (multi-set)	60 → 80% uninvolved limb	+10% every 2 weeks
Milandri & Sivarasu (2021) [21]	Mid	Eccentric cycling	2–3×/week	~3–4 × 8–12 min blocks	Submaximal (RPE)	Load via resistance control
Kasmi et al. (2023) [23]	Mid (~14 weeks)	ECC vs. PLYO vs. COMB	2×/week	~2–3 sets × multiple exercises	Moderate–high	Progressive overload
Kasmi et al. (2021) [22]	Mid (~14 weeks)	ECC vs. PLYO vs. COMB	2×/week	~2–3 sets	Moderate–high	Progressive overload
Stojanović et al. (2023) [29]	Mid–late	Flywheel	2–3×/week	2 × 6 → 3 × 10	High inertia (0.075 kg·m^2^)	Volume progression
Ong et al. (2024) [17]	Early–mid	Isokinetic eccentric	2–3×/week	3–4 × 6–10 reps	60–180°/s	Velocity-based
Coury et al. (2006) [26]	Late	Isokinetic eccentric	2×/week	3 × 10 reps	30°/s maximal	Constant
Brasileiro et al. (2011) [30]	Late	Isokinetic eccentric	2×/week	3 × 10 reps	30°/s maximal	Constant
Henderson et al. (2022) [19]	Late	Flywheel Bulgarian squat	2×/week	1 set to failure	0.025–0.075 kg·m^2^	Inertia progression
Norte et al. (2025) [28]	Late	Nordic hamstring	2×/week	3 × 6–10 reps	Bodyweight	Increasing reps

Abbreviations: ECC, eccentric training group; PLYO, plyometric training group; COMB, combined training group; MVC, maximal voluntary contraction.

**Table 5:** Methodological quality of included randomized controlled trials (PEDro scale).

Study	Random Allocation	Allocation Concealed	Baseline Comparability	Blinding Subjects	Blinding Therapists	Blinding Assessors	Follow-Up > 85%	Intention-to-Treat	Between-Group Comparison	Point Estimates & Variability	Total Score (/10)
Milandri & Sivarasu (2021) [21]	1	1	1	0	0	0	1	0	1	1	6
Vidmar et al. (2020) [18]	1	0	1	0	0	1	1	0	1	1	6
Patra et al. (2025) [31]	1	1	1	0	0	1	1	0	1	1	7
Gerber et al. (2007) [25]	1	0	1	0	0	1	1	0	1	1	6
Norte et al. (2025) [28]	1	1	1	0	0	1	1	0	1	1	7
Stojanović et al. (2023) [29]	1	0	1	0	0	1	1	0	1	1	6
Kasmi et al. (2023) [23]	1	0	1	0	0	1	1	0	1	1	6
Kasmi et al. (2021) [22]	1	0	1	0	0	1	1	0	1	1	6
Naczk et al. (2026) [20]	1	1	1	0	0	1	1	0	1	1	7

**Table 6:** Risk-of-bias assessment of non-randomized studies (ROBINS-I).

Study	Confounding	Selection of Participants	Classification of Intervention	Deviations from Intervention	Missing Data	Measurement of Outcomes	Reporting Bias	Overall Risk
Ong et al. (2024) [17]	Moderate	Moderate	Low	Moderate	Low	Low	Low	Moderate
Coury et al. (2006) [26]	Moderate	Moderate	Low	Moderate	Low	Moderate	Moderate	Moderate
Lepley et al. (2014) [27]	Moderate	Moderate	Low	Moderate	Low	Low	Moderate	Moderate
Henderson et al. (2022) [19]	Moderate	Moderate	Low	Moderate	Low	Low	Low	Moderate
Brasileiro et al. (2011) [30]	High	Moderate	Low	Moderate	Low	Moderate	Moderate	High

## Data Availability

No new data were created or analyzed in this study.

## Supplementary Materials

The following supporting information can be downloaded at: https://www.mdpi.com/article/10.3390/medicina62061109/s1. Supplementary S1: PRISMA 2020 Main Checklist [32]; Supplementary S2: Detailed Search Strategy.

## Abbreviations

The following abbreviations are used in this manuscript:

ACL	Anterior cruciate ligament
ACLR	Anterior cruciate ligament reconstruction
RFD	Rate of force development
NMES	Neuromuscular electrical stimulation
RTS	Return to sport
