# Supplementary material for: Eccentric-Oriented Strength Training in Anterior Cruciate Ligament Rehabilitation: A Scoping Review
Source: Medicina (Kaunas). 2026 Jun 7;62(6):1109. doi: 10.3390/medicina62061109 (PMC13303156; doi:10.3390/medicina62061109)
Supplement: Supplementary file 1 [file medicina-62-01109-s001.zip › medicina-4267002-supplementary-S2.pdf]

Supplementary Material S2.

Detailed Search Strategy

PubMed:
("anterior cruciate ligament"[Title/Abstract] OR "ACL"[Title/Abstract] OR "anterior cru-ciate ligament reconstruction"[Title/Abstract] OR "ACLR"[Title/Abstract]) AND ("eccentric training"[Title/Abstract] OR "eccentric exercise"[Title/Abstract] OR "eccentric strength training"[Title/Abstract] OR "eccentric loading"[Title/Abstract] OR "flywheel train-ing"[Title/Abstract] OR "isoinertial training"[Title/Abstract]).

Scopus:

TITLE-ABS ("anterior cruciate ligament" OR ACL OR "anterior cruciate ligament reconstruction" OR ACLR)
AND
TITLE-ABS ("eccentric training" OR "eccentric exercise" OR "eccentric strength training" OR "eccentric loading" OR "flywheel training" OR "isoinertial training")

Web of Science:

TS=("anterior cruciate ligament" OR ACL OR "anterior cruciate ligament reconstruction" OR ACLR)
AND
TS=("eccentric training" OR "eccentric exercise" OR "eccentric strength training" OR "eccentric loading" OR "flywheel training" OR "isoinertial training")

PEDro:

Search terms included: "ACL", "anterior cruciate ligament", and "eccentric".
Filters: clinical trials.

Limits applied:
English language only

Last search date:
22 February 2026
